# hFRUIT: An optimized agent for optical clearing of DiI-stained adult human brain tissue

**DOI:** 10.1038/s41598-020-66999-3

**Published:** 2020-06-19

**Authors:** Sven Hildebrand, Anna Schueth, Klaus von Wangenheim, Christian Mattheyer, Francesco Pampaloni, Hansjürgen Bratzke, Alard F. Roebroeck, Ralf A. W. Galuske

**Affiliations:** 1 Department of Cognitive Neuroscience, Faculty of Psychology & Neuroscience, Maastricht University, 6229 EV Maastricht, the Netherlands; 2 Department of Systemic Neurophysiology, Technische Universität Darmstadt, Schnittspahnstraße 3, Darmstadt, 64287 Germany; 3 Johann Wolfgang Goethe-Universität Frankfurt am Main, Faculty 15 Biological Sciences, Institute for Cell Biology and Neuroscience, Buchmann Institute for Molecular Life Sciences, Max-von-Laue-Str. 15, Frankfurt am Main, D-60438 Germany; 4 Johann Wolfgang Goethe-Universität Frankfurt am Main, Institute for Forensic Medicine, Kennedy Allee 104, 60596 Frankfurt am Main, Germany

**Keywords:** Neural circuits, Brain

## Abstract

Here, we describe a new immersion-based clearing method suitable for optical clearing of thick adult human brain samples while preserving its lipids and lipophilic labels such as 1,1′-dioctadecyl-3,3,3′,3′-tetramethylindocarbocyanine perchlorate (DiI). This clearing procedure is simple, easy to implement, and allowed for clearing of 5 mm thick human brain tissue samples within 12 days. Furthermore, we show for the first time the advantageous effect of the Periodate-Lysine-Paraformaldehyde (PLP) fixation as compared to the more commonly used 4% paraformaldehyde (PFA) on clearing performance.

## Introduction

The rediscovery of optical tissue clearing has attracted a lot of attention in neurobiological research during the last decade. In particular, for studies of the microcircuitry within and between cortical areas, optical clearing in combination with fluorescence microscopy offers new approaches as the very tedious and time consuming procedure of axonal reconstruction over large numbers of thin serial sections can be avoided. Numerous clearing protocols have been described and they can be grouped into only a few basic classes of clearing approaches^1,2^. Most of these protocols aim at an ever-increasing clearing capacity by removing the lipids from the tissue. To achieve this, they typically use either organic solvents, detergents or both^3^. While such protocols can clear big specimens, they are generally incompatible with lipophilic tracers such as dialkylcarbocyanine and dialkylaminostyryl dyes^4^. However, when it comes to studies on human *post mortem* tissue probes, lipophilic dyes are currently the only fluorophores allowing investigating microcircuitry at the level of individual neurons and axons which innervate specific regions of interest^5^. While immunofluorescent stainings of e.g. myelin or dendritic fibres allow for comparison on entire fibre populations, insertion of lipophilic dye crystals is currently still the best method available for human tissue, in order to selectively label neuronal processes innervating a specific region of interest. Similarly, there is no immunofluorescent label which can exclusively highlight neuronal cell bodies in the context of their connections to particular regions and layers. The ability of lipophilic dyes for characterizing cortical microcircuitry is therefore unique and a combination of these with optical clearing techniques in human tissue highly desirable. Generally, there are two different approaches to combine lipophilic dyes and optical clearing: The first one involves the use of fixable variants of commonly used lipophilic dyes and an additional fixation after staining which then preserves the staining after delipidation^6^. The second approach aims at clearing tissue samples without delipidation in an aqueous environment. This is a favourable procedure, because labelling with 1,1′-dioctadecyl-3,3,3′,3′-tetramethylindocarbocyanine perchlorate (DiI) and its derivatives is notoriously difficult in human *post mortem* material. Even chemically similar dyes like 3,3′-Dioctadecyloxacarbocyanine Perchlorate (DiO) show inferior diffusion properties in *post mortem* tissue^7^ and it is currently not known, if the fixable versions are suitable for human material at all. Only a few lipid-preserving protocols have been published so far and their clearing capacities are inferior to those of delipidating protocols. Among the first of the lipid preserving clearing protocols were the urea-based Sca*l*e^8^ and approaches based on sucrose^9^ or fructose^10,11^. The FRUIT protocol^12^, a combination of Sca*l*e^8^ and the fructose-based SeeDB^10,11^, is compatible with lipophilic tracers and exhibits reasonable clearing results, at least in mouse and rabbit brains. However, one important disadvantage of SeeDB and FRUIT is the Maillard^13^ reaction between the reducing sugar and tissue proteins, leading to profound tissue browning. Partly due to the Maillard reaction and partly due to the higher lipid content of the tissue, the abovementioned protocols deliver poor results in adult human brain tissue and human results have not yet been reported. Newer generations of simple immersion-based clearing protocols use other chemicals with high refractive index (RI) for clearing and are compatible with DiI-staining. However, these protocols are so far either tested in mouse or are limited to depths of about 1 mm in their clearing capacity in human brain tissue^4,14–17^.

To overcome all these obstacles we developed hFRUIT, an optimized version of the original FRUIT protocol, which we tested on thick DiI-labelled tissue samples from different species with high myelin content including human *post mortem* samples.

## Materials and Methods

### Human tissue

For DiI-labelling, fresh tissue samples containing the amygdala were dissected after autopsy from the brain of a 39 year old male without any known neurological disorders who had died from a perforating neck wound. The tissue was obtained with a *post mortem* delay of about 12 h. These samples were not fixed with formalin, but treated as described below (see “Tissue fixation and preparation for DiI application”). Tissue acquisition was approved by the local ethical committee of the Frankfurt University hospital.

Additionally, neocortical tissue samples were taken from human body donors (no known neuropathological diseases) of the body donation program of the Department of Anatomy and Embryology, Maastricht University. These samples were used for macroscopical investigation of the clearing efficacy of hFRUIT in standard formalin fixed brain samples. Donor tissue has also been used for Sudan Black B staining as described below (see “Comparison of lipid content between FRUIT and hFRUIT with Sudan Black B staining”). The tissue donors gave their informed and written consent to the donation of their body for teaching and research purposes as regulated by the Dutch law for the use of human remains for scientific research and education (“Wet op de Lijkbezorging”). Accordingly, a handwritten and signed codicil from the donor posed when still alive and well, is kept at the Department of Anatomy and Embryology Faculty of Health, Medicine and Life Sciences, Maastricht University, Maastricht, The Netherlands.

These brains were first fixed *in situ* by full body perfusion via the femoral artery. Under a pressure of 0.2 bar the body was perfused by 10 l fixation fluid (1.8 vol% formaldehyde, 20% ethanol, 8.4% glycerine in water) within 1.5–2 hours. Afterwards, the body was preserved for at least 4 weeks for post-fixation submersed in the same fluid. Subsequently, brains were recovered by calvarian dissection and stored in 4% paraformaldehyde (PFA) in 0.1 M phosphate buffered saline (PBS) for 14–30 months.

All methods were carried out in accordance with the relevant guidelines and regulations and all experimental protocols were approved by be Ethics Review Committee Psychology and Neuroscience (ERCPN).

For an overview of the different tissue sources and fixation protocols used in every figure presented, see Supplementary Table 1.

### Porcine tissue

Our initial experiments were conducted in fresh porcine brain tissue to ensure prudent use of scarce human brain tissue. The fresh tissue was received from a slaughterhouse immediately after sacrificing the animals. Samples were then immersion-fixed as described below.

### Murine tissue

For comparison of the clearing performance, brains of adult (4–5 months) B6.Cg-Tg(Thy1-YFP)HJrs/J mice (Jackson Laboratory) were used. The tissue was provided by the Department for Pharmacology and Toxicology of Maastricht University. The mice were bred under breeding license number B2015–003 and all brains provided were from mice from the breeding colonies, sacrificed as part of the breeding plan (surplus mice from breeding). Animals were sacrificed by CO_2_ inhalation in accordance with the EU guideline and the local animal regulations. Brains were collected directly and immersion fixated in 4% PFA in 0.1 M PBS at pH 7.4 for 24 h before being transferred to 0.1 M PBS containing 0.1% sodium azide. Brains were then cut into 3 mm thick coronal sections for clearing or processed without further dissection.

### Tissue fixation and preparation for DiI application

For DiI-labelling, both fresh porcine and the fresh human amygdala samples which had been dissected after autopsy (see first paragraph of the method section), were immediately fixed in Periodate-Lysine-Paraformaldehyde (PLP) fixative^18^. This fixative substantially facilitates the diffusion of DiI and is considered to be important for a high-quality DiI-labelling in human *post mortem* tissue, as first shown by Burkhalter and Bernado^19,20^. The fixative consists of 0.1 M PBS at pH 7.4, 2.6% PFA, 0.8% iodoacetic acid, 0.8% sodium periodate, and 0.1 M D-L-lysine and the samples were fixed prior to labelling for 2–7 days. After labelling, samples were kept in 2% PFA in 0.1 M PBS at pH 7.4 for 3 months (porcine samples) or at least 6 months up to several years (human samples) at 37 °C in the dark, with the fixative being replaced regularly.

### Post mortem axonal tracing with DiI

After the initial PLP fixation for 2–7 days described above, samples were washed in 0.1 M PBS. For DiI labelling, a small incision was made in the grey matter of the tissue blocks and crystals of the dye (Thermo Fisher Scientific Inc., Waltham, Massachusetts, USA) were placed into the tissue using an ethanol filled glass pipette with a tip diameter of approx. 50 µm. Special care was taken to avoid direct contact of the dye crystals with the white matter.

### Optimization of hFRUIT clearing for human brain tissue

In our new clearing protocol, we increased 1-thioglycerol (M1753, Sigma-Aldrich Corp., St. Louis, Missouri, USA) concentrations to minimize the Maillard-reaction caused by high fructose concentrations. Moreover, 1-thioglycerol has a high refractive index (RI) and, therefore, a positive effect on the clearing performance. In contrast to previous protocols, which exclusively used fructose, the fructose concentration was reduced in hFRUIT and compensated with the non-reducing sugar sucrose (Merck KGaA, Darmstadt, Germany). This further minimizes the Maillard-reaction. In order to optimize the composition of the different components of the clearing solution, the content of the different components was systematically varied (see Table 1). All clearing steps were carried out at room temperature on a shaker. For an accelerated dissolution of the 80% and 100% solutions, fructose, sucrose, and 1-thioglycerol should be mixed under stirring in a small volume of distilled water at 60–70 °C until completely dissolved. The solution must cool down before adding urea to avoid its decomposition and distilled water is added up to the final volume for the respective concentration. All samples were incubated in 50 ml tubes filled with the solution. Before the immersion in the final 100% hFRUIT solution, samples were carefully blotted on paper tissues and new 50 ml tubes were used, to avoid contamination of the lower concentrated solution. The final RI of the hFRUIT solution was between 1.49–1.50 and thus very close to that of immersion oil (1.52).

**Table 1:** Concentrations and incubation times for the hFRUIT clearing protocol.

Incubation time [days]	Urea [g/ml]	Fructose [g/ml]	Sucrose [g/ml]	Thioglycerol [g/ml]
1	0.24	0.04	0.04	0.02
1	0.24	0.08	0.08	0.04
2	0.24	0.16	0.16	0.08
2	0.24	0.24	0.24	0.12
3	0.24	0.32	0.32	0.16
3	0.24	0.385	0.37	0.2

The times indicated here have been used for all the samples used in this study. However, incubation times depend on samples size and have to be determined empirically and smaller samples are expected to clear faster.

### FRUIT clearing of the brain tissue

At first, FRUIT clearing was performed according to the original publication^12^. Samples were incubated in ascending concentrations of FRUIT solution containing 20%, 40%, 60%, 80% and 100% (w/v) fructose respectively. All solutions contained 0.5% (w/v) 1-thioglycerol and 24% (w/v) urea and were dissolved in distilled water. Incubation was carried out for 8 h (20–60%), 12 h (80%), and 24 h (100%) at 37 °C. Because of the intensive tissue-browning due to the Maillard reaction observed at these elevated temperatures, FRUIT was rather carried out at room temperature (RT). For better comparison with the hFRUIT protocol, the same incubation times were used on both murine and porcine brain samples cleared with either FRUIT or hFRUIT as described above: 1 day in 20%, 2 days each in 40% and 60%, and 3 days each in 80% and 100% (see also Table 1). All samples were incubated on a shaker in 50 ml tubes filled with the solution.

### Comparison of lipid content between FRUIT and hFRUIT with Sudan Black B staining

Human neocortex samples obtained from body donors, which have been perfusion fixed in formalin and post-fixed in 4% PFA, were taken from the same gyrus and either treated with hFRUIT as described above or stored in 0.1 M PBS with 1% sodium azide as a control. After clearing, the samples were immersed again in 0.1 M PBS and cut into 50 µm sections on a vibratome (VT1200 S, Leica Biosystems GmbH, Wetzlar, Germany). Sections were mounted on gelatinized glass slides and air dried overnight. Then, sections were washed in distilled water for 5 min, dehydrated in 70% ethanol for 5 min and stained in 0.1% Sudan Black B (Merck KGaA, Darmstadt, Germany) in 70% ethanol for 10 min. Excess dye was washed out in 70% ethanol and distilled water for 5 min each before cover-slipping in Kaiser’s glycerol gelatine (Carl Roth GmbH & Co. KG, Karlsruhe, Germany).

### Confocal laser scanning microscopy of porcine brain samples

Samples were imaged on a LSM 780 Axio Observer (Carl Zeiss AG, Jena, Germany) equipped with a Plan-Apochromat 10x/0.3 M27 objective. For imaging of autofluorescent vasculature, 488 nm excitation and a detection wavelength between 493 and 552 nm was chosen. For imaging of the DiI signal, 514 nm was used for excitation and a detection band of 546–672 nm.

### Light-sheet microscopic imaging of human brain samples

Before imaging, samples were taken out of the 100% hFRUIT solution and carefully blotted dry with paper towels. After that the samples were immersed in a 1:1 mixture of mineral oil (M8410, Sigma-Aldrich Corp., St. Louis, Missouri, USA) and silicone oil (175633, Sigma-Aldrich Corp., St. Louis, Missouri, USA). This solution has the same RI as the clearing solution but is much less viscous and allows easier handling. Imaging was performed on a diSPIM set-up (Applied Scientific Instrumentation Inc., Eugene, Oregon, USA) with objectives tuneable to RIs of 1.33–1.56, resulting in a numerical aperture (NA) of 0.37–0.43 and 15.3x–17.9x magnification respectively (Special Optics Inc., Denville, New Jersey, USA). For excitation, a 561 nm laser-line was used.

### Comparison of imaging depth

For the comparison of the clearing capacity of FRUIT and hFRUIT, 6 porcine brain samples for each clearing approach were imaged on a confocal microscope as described above and z-stacks with a step size of 35 µm of the autofluorescent signal of blood vessels in 3 regions of interest (ROIs) per sample were acquired. Subsequently, the clearing capacity of each protocol was quantified on the unprocessed image files. To this end, the maximally achievable imaging depth was assessed by the contrast decay of the median pixel intensity value per plane in the acquired stacks for both clearing protocols as has been described earlier by Costantini *et al*.^21^. The distribution of measured imaging depths of FRUIT and hFRUIT cleared samples was tested for normal distribution with a Kolmogorov-Smirnov test as well as an Anderson-Darling test. For comparison between FRUIT and hFRUIT cleared samples, an unpaired t-test was used.

### Data processing

The data sets acquired with the light sheet microscope were processed with Huygens Professional (v19.10; Scientific Volume Imaging B.V., Hilversum, the Netherlands). The raw data was first deskewed using the light sheet deskewing mode in the Huygens Object Stabilizer. The point spread function was extracted from the deskewed dataset using the PSF distiller and the data was subsequently deconvolved with the extracted PSF using the Huygens CMLE algorithm (40 iterations, SNR 40, manual background). Maximum intensity projections or 3D renderings were also generated with Huygens Professional. For all other processing steps of the images such as brightness and contrast adjustments or denoising, the open-source platform FIJI was used^22^. For the assessment of imaging depth as described above, a custom-made script in MATLAB R2015a (The MathWorks, Inc., Natick, Massachusetts, USA) was used. Statistical analysis was likewise performed in MATLAB.

## Results

### Optimization of the hFRUIT protocol

The optimal parameters for the hFRUIT protocol are given in Table 1. Adding the non-reducing sugar sucrose to the solution increased the clearing capacity. Surprisingly, exchanging fructose entirely with sucrose did not produce markedly better clearing results as compared to the sucrose/fructose mixture, even though the Maillard reaction should have been minimized. This is probably due to the fact that in the mixture a higher amount of sugar can be dissolved in total, which further increases the RI. Nevertheless, the solution containing only sucrose as sugar displayed less tissue browning and improved transparency as compared to the original fructose based FRUIT protocol.

The biggest effect on the enhancement of clearing performance however, was the increase of the 1-thioglycerol concentration from 0.5% (w/v) as in the original protocol to 20% (w/v) as a final concentration. This concentration was the highest possible for 1-thioglycerol, because the dissolution of urea and sugars is no longer possible above the concentration of 20% (w/v).

### Comparison of FRUIT and hFRUIT in murine and porcine brain tissue samples

**Figure 1:**
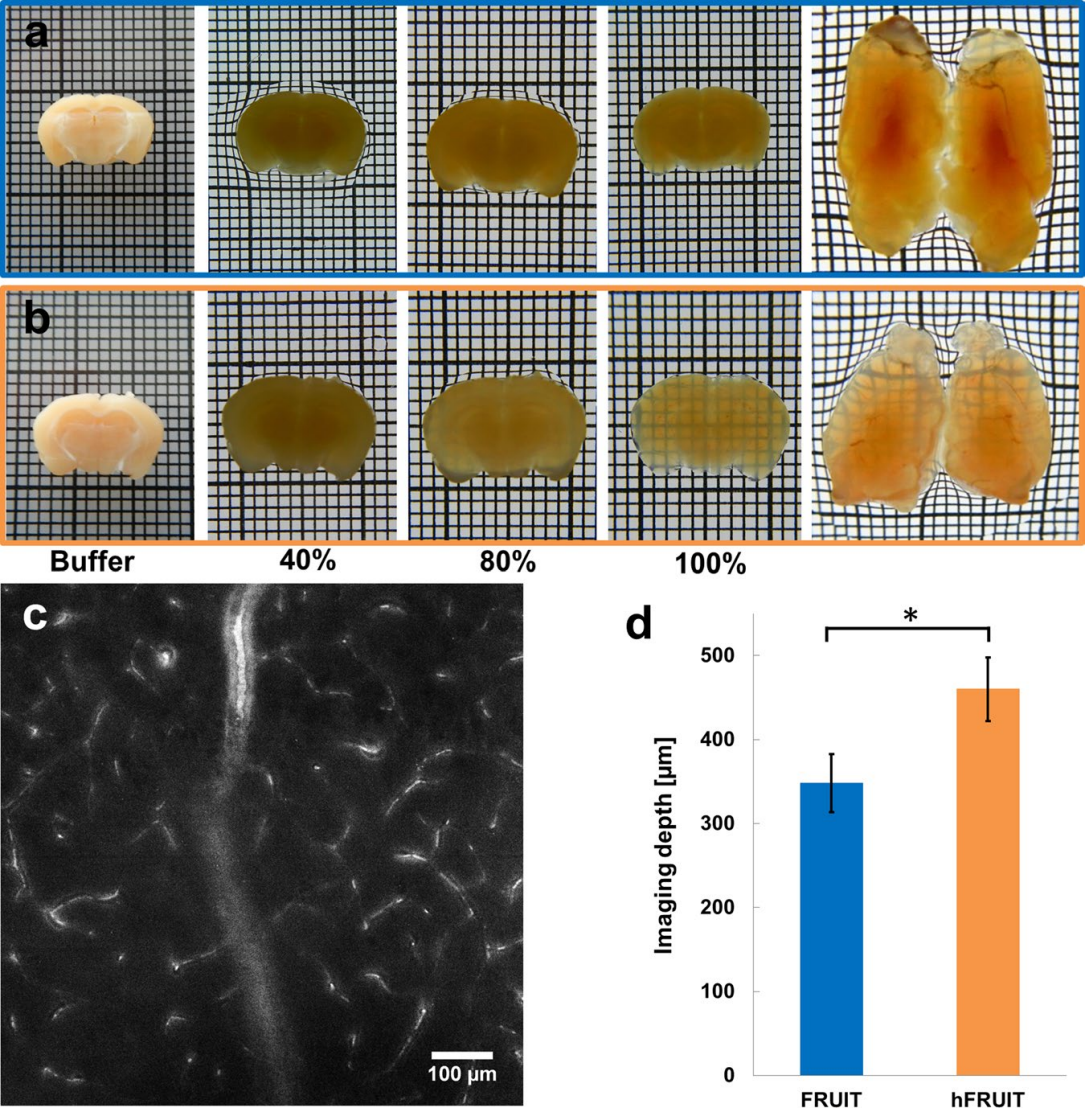
Comparison of the clearing capacity of FRUIT and hFRUIT on non-human brain samples. Comparison of murine brain samples cleared with FRUIT (**a**; blue) and hFRUIT (**b**; orange). The first four panels in a and b show 3 mm thick coronal brain sections at varying points during the clearing process. The last panels in a and b show two whole hemispheres after clearing. The heavily myelinated regions in the medial and posterior parts of the brain are generally more difficult to clear without delipidation. (grid: 1 × 1 mm). (**c**) typical example of autofluorescent background with 488 nm excitation in a 3 mm thick porcine brain sample cleared with hFRUIT at a depth of 315 µm at 10x magnification. (**d**) contrast decay of autofluorescent signal as function of depth (Costatini *et al*., 2015) in porcine samples cleared with FRUIT (blue) or hFRUIT (orange; n = 18 respectively; error bars indicate SEM; unpaired t-test: p = 0,0361).

In order to compare the clearing capacity of our new solutions both, 3 mm thick coronal sections and entire mouse brains were cleared. Upon macroscopic inspection, samples treated with hFRUIT appeared much more transparent as compared to the original protocol (Fig. 1a,b, Supplementary Fig. 1). This was mainly due to reduced tissue browning. In order to quantify this difference and to test the clearing capacity on larger and more myelinated tissue, 3 mm thick porcine brain sections were cleared with either method and the autoflourescent signal of cortical vasculature was imaged (Fig. 1c). The maximally achievable imaging depth of both methods, was assessed by comparing the contrast decay of the median pixel intensity value per plane in the acquired stacks as has been described earlier by Costatini *et al*.^21^. The average depth to which samples could be imaged was found to be greater in tissue cleared with hFRUIT (unpaired t-test: p = 0.0361), despite the relatively short excitation wavelength of 488 nm, confirming our observations in the murine samples (Fig. 1d).

### Preservation of DiI label after clearing

**Figure 2:**
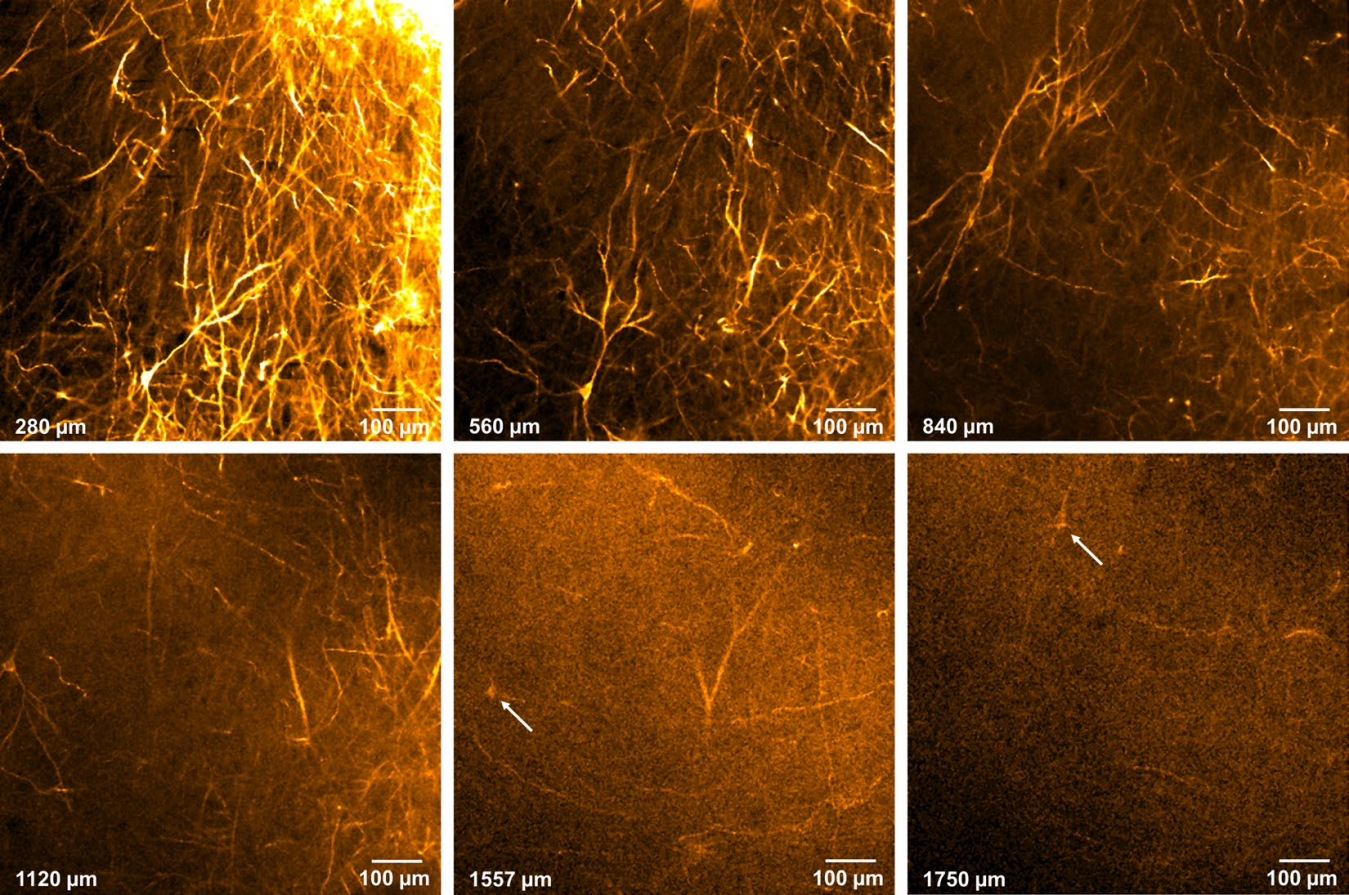
DiI stained neurons and neuronal processes in a porcine brain sample cleared with hFRUIT. Panels show different planes from the same z-stack imaged with CLSM (10x magnification). Despite the increasing background over depth, cell bodies and processes are visible up to at least 1500 µm (white arrows).

Before applying the hFRUIT protocol to human brain tissue samples, we tested whether the DiI label is preserved after our clearing procedure. To this end 3 mm thick porcine brain samples were incubated with DiI crystals for 3 months and then cleared and imaged using an inverted confocal microscope. As shown in Fig. 2 and Supplementary Fig. 2, the label was well preserved after clearing and the longer exitation wavelength allowed for even deeper imaging as compared to the wavelength used for the autofluorescence imaging. Individual cell bodies and fibers were clearly discernable at depths up to 1.5 mm and more.

### Capacity of hFRUIT for clearing human brain tissue

**Figure 3:**
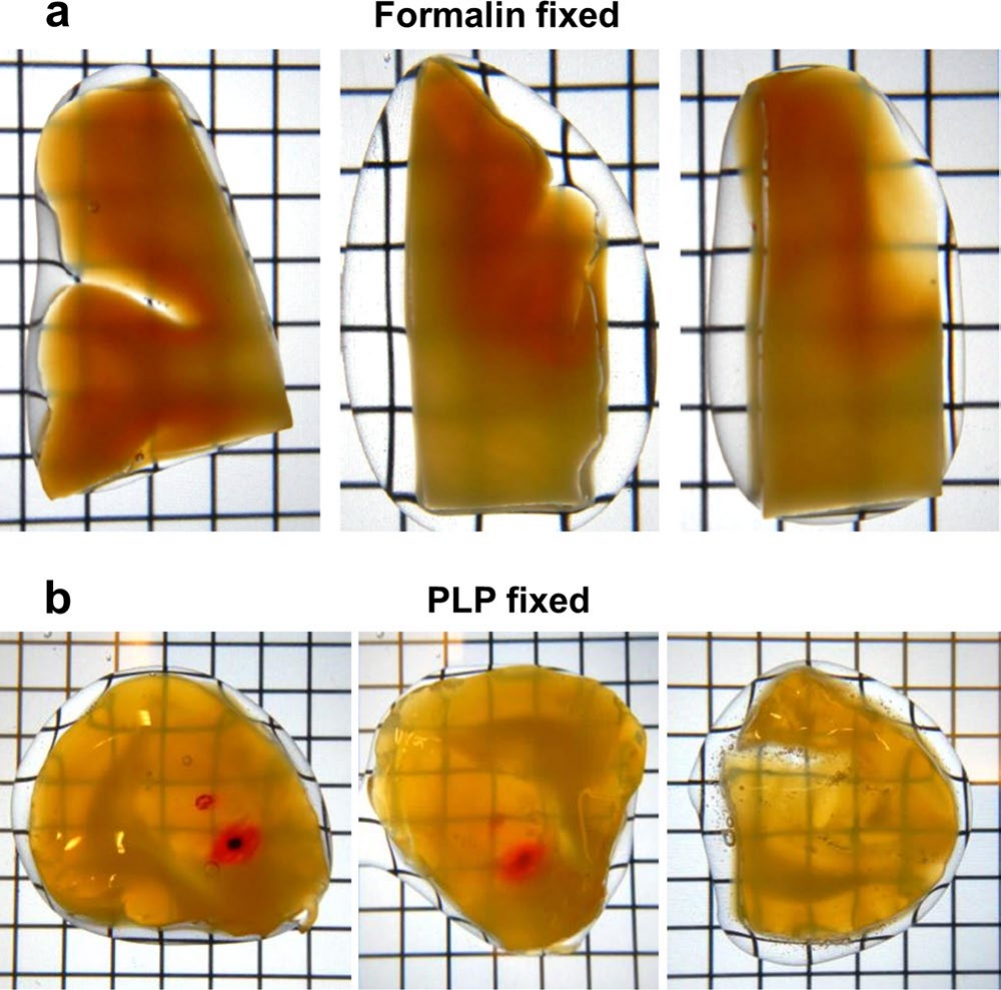
Effect of fixative on clearing capacity with hFRUIT. Transparency of 5 mm thick human neocortex samples fixed with formalin is limited (**a**). When samples are fixed with Burkhalter and Bernado’s variation of the PLP-fixation (**b**; Burkhalter and Bernado, 1989) the transparency is considerably improved, as shown here on 5–7 mm thick human amygdala samples. Note that both injection sites are clearly visible from the backside through the entire thickness of the sample in the left and middle panel. In this sample the thickness varies from about 7 mm (on the left side in left panel) and 5 mm on the opposite side. The second sample has a more even thickness of about 5 mm. Grid: 5 × 5 mm.

First, the clearing capacity of hFRUIT was tested on unlabelled human neocortical samples of 5 mm thickness fixated with formalin. As expected, the clearing in grey matter was better as compared to white matter, as the difference in RI between white and grey matter cannot be alleviated without delipidation (Fig. 3a). When the same clearing method was used on DiI-labelled amygdala samples treated with the PLP fixation, the transparency was superior for grey matter regions (Fig. 3b). The first sample was wedge shaped and approximately 7 mm thick on one side (left side of the tissue in the first panel of Fig. 3b), and about 5 mm thick on the other side (right side in Fig. 3b first panel). The second sample was more evenly cut at about 5 mm thickness and appears a bit lighter overall because of this. However, even on thinner tissue regions, the heavily myelinated white matter rendered parts containing a lot of fibre bundles more opaque, as the abundance of lipids changes the RI in the respective tissue regions.

**Figure 4:**
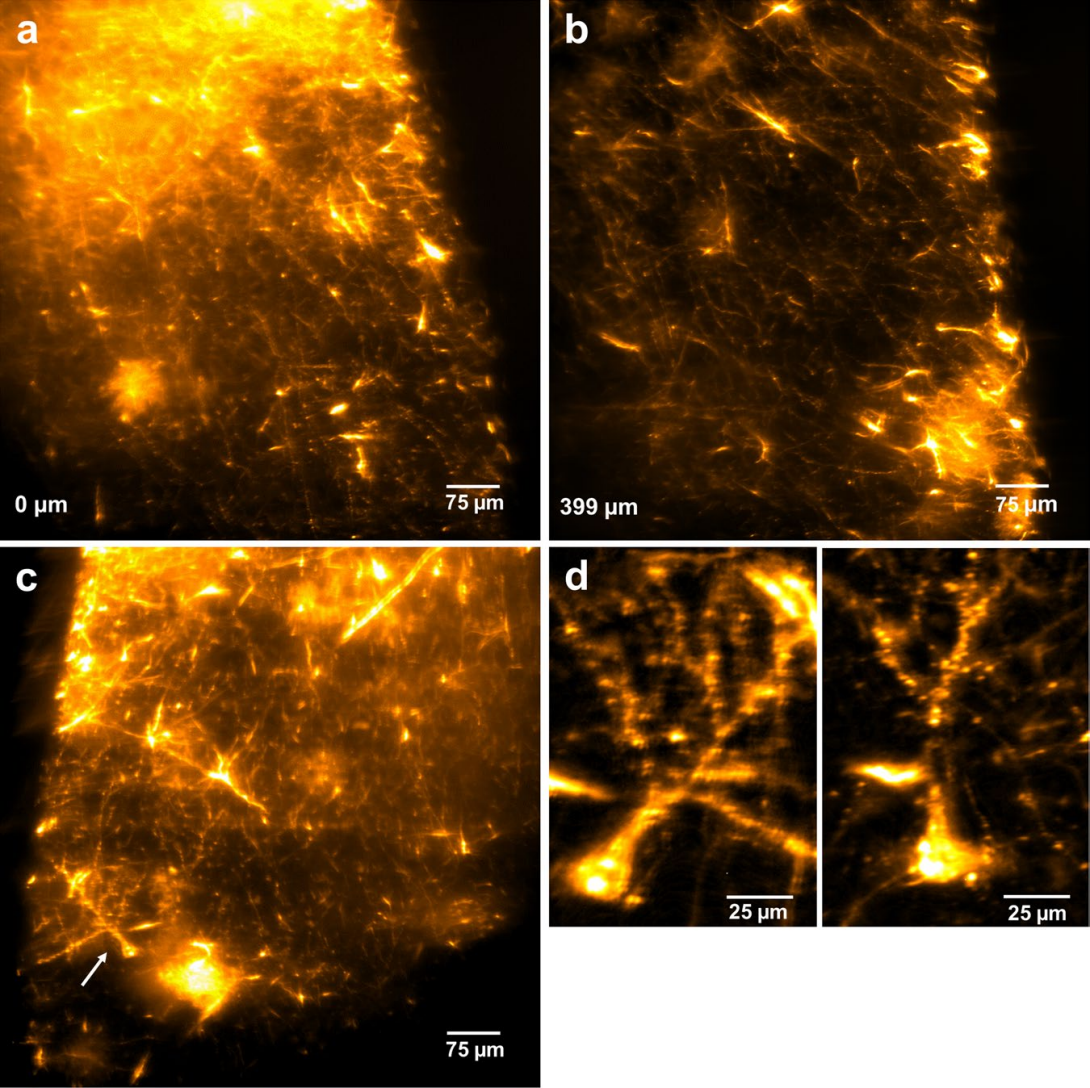
Light sheet microscopy of DiI-stained human amygdala. (**a**) The DiI label in close proximity to the injection site of the crystal (upper part of the image). (**b**) Label of individual fibres 400 µm lateral from the injection site was well preserved. (**c**) The staining quality after clearing was good enough to clearly distinguish labelled neurons (arrow). (**d**) Enlarged maximum intensity projections over 10 µm of two labelled neurons with dendrites including spine-like protrusions clearly visible.

**Figure 5:**
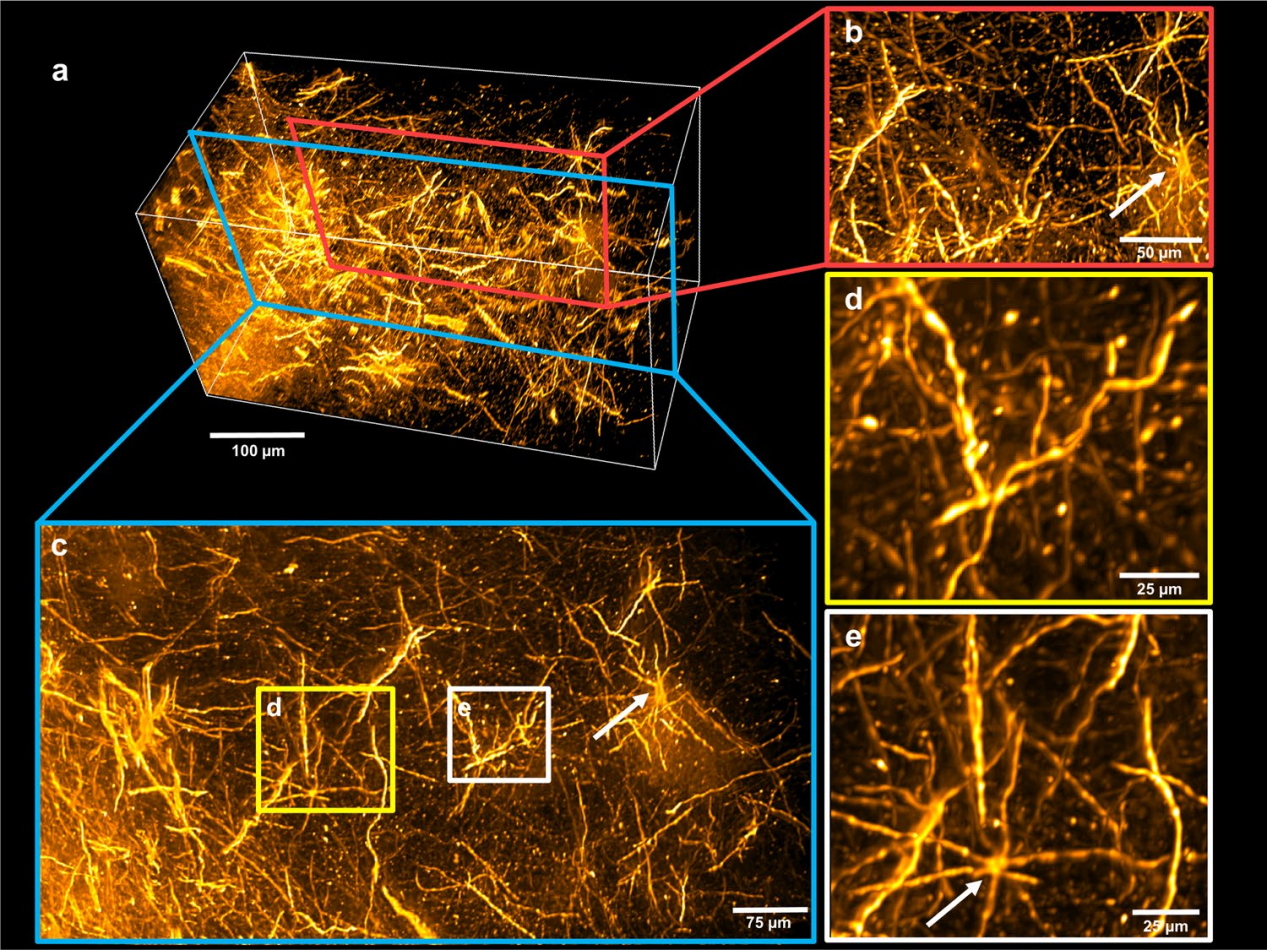
Deconvolved light sheet data. (**a**) 3D rendering of a sub-volume of one view. (**b**) Magnified view of a part of the rendering showing cell bodies (arrow) and fibres throughout the field of view. (**c**) Maximum intensity projection over 60 µm of the volume. (**d**,**e**) Enlarged regions of the projection showing neuron soma (arrow) and good contrast of even the smallest stained nerve fibers.

The human amygdala samples were imaged on a diSPIM system (Fig. 4, Supplementary Video 1–2). Images were acquired in the vicinity of the injection site of the DiI crystal (bright region in upper part of Fig. 4a,c). The preservation of the label in human tissue was excellent and provided a strong contrast of individual fibres to the background (Fig. 4b). Whole cells could be observed, including their dendrites with protrusions which might be indicative of spines (Fig. 4c,d). The volume was acquired as an x-scan over a region of 400 × 772 × 546 µm starting in close proximity to the injection site. A single view dataset was taken for further processing with the Huygens software for deconvolution. After this steps, individual fibres could be easily identified in 3D renderings of a sub-volume from all sides (Fig. 5a,b, Supplementary Video 3) and stood out clearly in maximum intensity projections over a depth of 60 µm (Fig. 5c–e).

**Figure 6:**
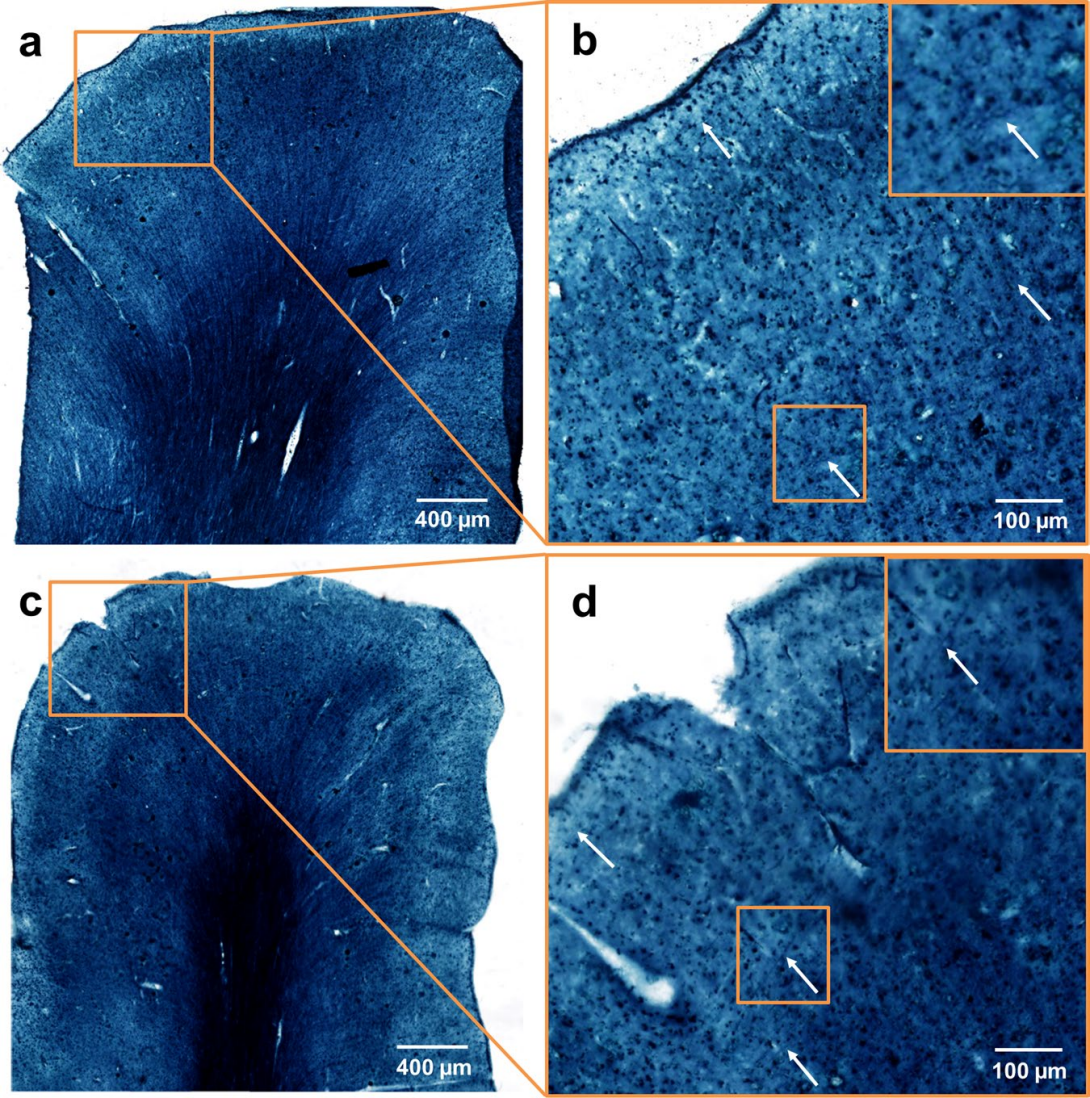
Sudan Black B staining for lipids on uncleared human brain sections (**a,b**) and sections of an hFRUIT cleared sample (**c,d**). For both conditions the abundance of lipids is evident by the dark blue-black staining. At lower magnification, the intensely stained white matter as well as radial fibre bundles are clearly distinguishable over a general light blue background (**a** and **c**). Likewise, in higher magnifications of ROIs in superficial cortical regions, individual tangential fibers can be detected in both cleared (**b**) and uncleared (**d**) sections (arrows in **b** and **d** and insets therein from indicated regions). The intensely stained small spots visible in the grey matter of both conditions are likely lipofuscin aggregates.

In order to access the lipid-preserving properties of our clearing technique, additional hFRUIT treated samples were rehydrated and 50 µm thick sections were cut with a vibratome. These sections were stained with Sudan Black B as a general lipid marker and compared to uncleared controls. Both groups were intensely stained both in white and grey matter (Fig. 6). As seen already with DiI, individual fibres in the grey matter remained well discernible within the light blue neuropil (white arrows in Fig. 6b,d and insets therein). The intensely stained spots visible throughout the grey matter of both groups are likely to be clusters of lipofuscin, a lipid containing pigment that accumulates over age as a product of lipid metabolism^23,24^. These findings demonstrate that our new clearing protocol leaves a substantial part of the brain lipid fraction intact and, thus, accessible for tracing techniques using lipophilic dyes. At the same time our new protocol is able to render highly myelinated tissues transparent over several millimetres, which makes it a highly promising approach for tracer studies in cleared adult human brain tissue.

## Discussion

The hFRUIT protocol allows for the first time clearing of fixed adult human brain tissue of several millimetre thickness while leaving the majority of lipids intact. The original FRUIT protocol already provided reasonable performance in murine and rabbit brain tissue, but the clearing capacity in higher mammals and especially human tissue was very limited with this type of clearing approaches^11,12,15,25,26^. Clearly, the higher myelin content of human brain tissue is the main reason for this. Even though delipidating clearing approaches might lead to better results in human brain tissue, such approaches cannot be used in circumstances which require preservation of lipids. One of the most important instances in this context is the targeted study of microcircuits. So far the use of lipophilic tracers such as DiI is the only approach to study microcircuitry in a targeted manner in the human brain. Even though fixable lipophilic dyes are available^6^, it is currently unknown whether these dyes have the same staining capacity as the traditional dyes such as DiI and DiA. The labelling procedure in human *post mortem* material with DiI crystals is notoriously difficult and modified fixable derivatives might not have the same diffusion properties in human tissue as compared to other mammalian brains. In our experience the best staining results depend on the following factors: a *post mortem* interval of less than 24 hours^27–30^, PLP fixation of the tissue^19,20,30^, and incubation at 37 °C in the dark to enhance diffusion.

Therefore, hFRUIT offers in principle a great advantage when aiming at tracing local circuits in the human brain on the level of individual axons. However, currently some aberrations can be observed in our data set which might limit the ability of accurate tracing. The aberrations occur mainly in the periphery of the image while in the centre, individual fibres are clearly visible (see Fig. 4, Supplementary Video 1–2). Thus, it is unlikely that these aberrations result from RI inhomogeneities between the tissue and the imaging solution, because these aberrations should be equally distributed throughout the imaging plane. It is more likely that aberrations result from small discrepancies in the calibration of the microscope set-up as the objectives used for imaging were prototypes (kindly provided for testing by Applied Scientific Instrumentation Inc., Eugene, Oregon, USA) and tested for the first time on tissue samples.

While the magnification of these objectives is enough to demonstrate single fibres, the clear visualization of dendritic spines is currently not unequivocally possible. Even though the protrusions observed on dendrites (Fig. 4d) might be indicative of spines, it cannot be excluded that they are results of blebbing due to apoptosis or tissue degradation in the *post mortem* brains before fixation. One possible way to address this issue would be the use of higher magnification objectives and simultaneous deconvolution of dual view data from both angles. In principle, the dual view deconvolution would allow for an even higher, near isotropic, resolution compared to the deconvolved single views shown in Fig. 5 and Supplementary Video 3. However, this would require very good spatial registration of the two views and the aberrations discussed above make a trivial linear registration of the two angles insufficient to perfectly align both views. Therefore, effective dual view deconvolution will require future work to focus on more advanced 3D image alignment routines. Future studies using a larger sample size and focusing on the data analysis aspect of the processing pipeline, would allow investigating the advantages of dual view deconvolved data versus single view deconvolution in a more appropriate manner. We are confident that an optimized data acquisition and post-processing, in combination with our clearing approach, will allow for complete 3D reconstruction over large volumes of DiI-labelled local neuronal networks. This enables circuitry visualization at a quality level which is so far only achievable on thin sections with traditional techniques, such as Golgi stains or intracellular fillings in human post mortem brain tissue^5^.

Much to our surprise, modification of the fixation protocol from a simple formalin fixation to the PLP fixation greatly enhanced the efficacy of hFRUIT (Fig. 3). Even though the PLP fixation had been chosen because it improves the DiI staining, it also improved the transparency obtained with hFRUIT. The reason for the enhanced clearing of samples fixed with the PLP fixation is currently unknown. However, it is unlikely that only the lower concentration of PFA is responsible for this finding. The long incubation times in fixative of at least three months should be sufficient to allow for complete fixation of the tissue, despite the lower PFA concentrations of 2.6% in the initial and 2% in the post-fixation^7^. Therefore, the beneficial effect must be due to either the different chemical composition of the tissue by preservation of other tissue components or by interactions between the compounds of the fixative and the clearing solution. We consider this observation to be of high interest. Future investigations should focus on testing alternative fixatives, such as the recently proposed glyoxal fixation^31^ or the PLP fixation used in this study in order to establish new standard fixatives for clearing and expansion microscopy^32–34^. Since these alternative solutions supposedly preserve the ultrastructure of the tissue much better than formalin or 4% PFA while retaining compatibility with e.g. immunohistochemistry they could be of particular interest for super-resolution microscopy on expanded samples^18,31,35^.

For future investigations using the hFRUIT approach, a simple counterstaining procedure is desirable to provide the relevant cytoarchitectural framework for the interpretation of the connections visualized with DiI. The recently developed MASH approach^36^ provides options for a suitable counterstaining, if the staining method is combined with hFRUIT clearing. Likewise, the reconstruction of the local circuitry revealed with hFRUIT and the tracing of these connections in 3D will be the focus of future investigations.

The combination with the above mentioned improvements will help to advance the understanding of the intrinsic cortical circuitry in humans, where the available range of techniques is considerably more limited as compared to animal models.

## Conclusions

We successfully developed hFRUIT, an improved immersion-based protocol capable of clearing human brain tissue while preserving its lipids and lipophilic dyes such as DiI. This makes it a great tool to easily study brain microcircuits in large human tissue blocks in three dimensions. The protocol is simple and achieved clearing of 5 mm thick human brain samples in 12 days. Furthermore, it is important to note that different fixatives seem to crucially influence the clearing performance. This is of great interest to the clearing community as it might allow for improving the performance of other clearing protocols as well. This holds not only for human tissue, but is also important for animal models especially as the choice of fixative may be more flexible as compared to human *post mortem* tissue.

## Supplementary information

Supplementary Video 1.

Supplementary Video 2.

Supplementary Video 3.

Supplementary information.

## Data Availability

The datasets generated and/or analyzed during the current study are available from the corresponding author on reasonable request.

## References

[1] Richardson DS, Lichtman JW (2017). SnapShot: Tissue Clearing. Cell.

[2] Richardson DS, Lichtman JW (2015). Clarifying Tissue Clearing. Cell.

[3] Tainaka K, Kuno A, Kubota SI, Murakami T, Ueda HR (2016). Chemical Principles in Tissue Clearing and Staining Protocols for Whole-Body Cell Profiling. Annu. Rev. Cell Dev. Biol..

[4] Chen L (2017). UbasM: An effective balanced optical clearing method for intact biomedical imaging. Sci. Rep..

[5] Lanciego JL, Wouterlood FG (2011). A half century of experimental neuroanatomical tracing. Journal of Chemical Neuroanatomy.

[6] Jensen KHR, Berg RW (2016). CLARITY-compatible lipophilic dyes for electrode marking and neuronal tracing. Sci. Rep..

[7] Chen BK (2006). Optimizing conditions and avoiding pitfalls for prolonged axonal tracing with carbocyanine dyes in fixed rat spinal cords. J. Neurosci. Methods.

[8] Hama H (2011). Scale: A chemical approach for fluorescence imaging and reconstruction of transparent mouse brain. Nat. Neurosci..

[9] Tsai PS (2009). Correlations of Neuronal and Microvascular Densities in Murine Cortex Revealed by Direct Counting and Colocalization of Nuclei and Vessels. J. Neurosci..

[10] Ke MT, Fujimoto S, Imai T (2013). SeeDB: A simple and morphology-preserving optical clearing agent for neuronal circuit reconstruction. Nat. Neurosci..

[11] Ke, M. T. & Imai, T. Optical clearing of fixed brain samples using SeeDB. *Curr. Protoc. Neurosci*. 10.1002/0471142301.ns0222s66 (2014). 10.1002/0471142301.ns0222s66 24510778

[12] Hou B (2015). Scalable and DiI-compatible optical clearance of the mammalian brain. Front. Neuroanat..

[13] Dills WL (1993). Protein fructosylation: fructose and the Maillard reaction. Am. J. Clin. Nutr..

[14] Aoyagi Y, Kawakami R, Osanai H, Hibi T, Nemoto T (2015). A rapid optical clearing protocol using 2,2′-thiodiethanol for microscopic observation of fixed mouse brain. PLoS One.

[15] Li, W., Germain, R. N. & Gerner, M. Y. Multiplex, quantitative cellular analysis in large tissue volumes with clearing-enhanced 3D microscopy (C _e_ 3D). Proc. Natl. Acad. Sci. 201708981 (2017). 10.1073/pnas.1708981114 10.1073/pnas.1708981114 PMC5584454 28808033

[16] Lai HM, Ng WL, Gentleman SM, Wu W (2017). Chemical Probes for Visualizing Intact Animal and Human Brain Tissue. Cell Chem. Biol..

[17] Yu T (2018). RTF: A rapid and versatile tissue optical clearing method. Sci. Rep..

[18] McLean IW, Nakane PK (1974). Periodate lysine paraformaldehyde fixative. A new fixative for immunoelectron microscopy. J. Histochem. Cytochem..

[19] Burkhalter A, Bernardo KL (1989). Organization of corticocortical connections in human visual cortex. Proc. Natl. Acad. Sci..

[20] Burkhalter A, Bernardo KL, Charles V (1993). Development of local circuits in human visual cortex. J. Neurosci..

[21] Costantini I (2015). A versatile clearing agent for multi-modal brain imaging. Sci. Rep..

[22] Schindelin J (2012). Fiji: An open-source platform for biological-image analysis. Nat. Methods.

[23] Schnell SA, Staines WA, Wessendorf MW (1999). Reduction of lipofuscin-like autofluorescence in fluorescently labeled tissue. J. Histochem. Cytochem..

[24] Gray DA, Woulfe J (2005). Lipofuscin and aging: a matter of toxic waste. Sci. Aging Knowledge Environ..

[25] Ke MT (2016). Super-Resolution Mapping of Neuronal Circuitry With an Index-Optimized Clearing Agent. Cell Rep..

[26] Lai, H. M. *et al*. Author Correction: Next generation histology methods for three-dimensional imaging of fresh and archival human brain tissues (Nature Communications (2018) 9 (1066) DOI: 10.1038/s41467-018-03359-w). *Nat. Commun*. 9, (2018). 10.1038/s41467-018-03359-w PMC5852003 29540691

[27] Ferrer I, Martinez A, Boluda S, Parchi P, Barrachina M (2008). Brain banks: Benefits, limitations and cautions concerning the use of post-mortem brain tissue for molecular studies. Cell Tissue Bank..

[28] Ferrer I (2007). Brain protein preservation largely depends on the postmortem storage temperature: Implications for study of proteins in human neurologic diseases and management of brain banks: A BrainNet Europe study. J. Neuropathol. Exp. Neurol..

[29] Crecelius A (2008). Assessing quantitative post-mortem changes in the gray matter of the human frontal cortex proteome by 2-D DIGE. Proteomics.

[30] Galuske RAW, Schlote W, Bratzke H, Singer W (2000). Interhemispheric asymmetries of the modular structure in human temporal cortex. Science (80-.)..

[31] Richter KN (2017). Glyoxal as an alternative fixative to formaldehyde in immunostaining and super‐resolution microscopy. EMBO J..

[32] Chen F, Tillberg PW, Boyden ES (2015). Expansion microscopy. Science (80-.)..

[33] Murakami TC (2018). A three-dimensional single-cell-resolution whole-brain atlas using CUBIC-X expansion microscopy and tissue clearing. Nat. Neurosci..

[34] Tillberg PW (2016). Protein-retention expansion microscopy of cells and tissues labeled using standard fluorescent proteins and antibodies. Nat. Biotechnol..

[35] Hall, P. A., Stearn, P. M., Butler, M. G. & D’ardenne, A. J. *Acetone/periodate-Iysine-paraformaldehyde (PLP) fixation and improved morphology of cryostat sections for immunohistochemistry*. *Histopathology* **11** (1987). 3030921

[36] Hildebrand, S., Schueth, A., Herrler, A., Galuske, R. & Roebroeck, A. *Scalable cytoarchitectonic characterization of large intact human neocortex samples*. *bioRxiv* 274985, 10.1101/274985 (2018). 10.1038/s41598-019-47336-9 PMC6659684 31350519

