# Supplementary material for: hFRUIT: An optimized agent for optical clearing of DiI-stained adult human brain tissue
Source: Sci Rep. 2020 Jun 19;10:9950. doi: 10.1038/s41598-020-66999-3 (PMC7305111; doi:10.1038/s41598-020-66999-3)
Supplement: Supplementary file 4 — Supplementary information. [file 41598_2020_66999_MOESM4_ESM.pdf]

Supplementary information:

hFRUIT: An optimized agent for optical clearing of DiI-stained adult human brain tissue

Sven Hildebrand[1,*], Anna Schueth[1], Klaus von Wangenheim[2], Christian Mattheyer[3], Francesco Pampaloni[3], Hansjürgen Bratzke[4], Alard F. Roebroeck[1], Ralf A. W. Galuske[2,*]

1. Department of Cognitive Neuroscience
Faculty of Psychology & Neuroscience
Maastricht University
6229 EV Maastricht
the Netherlands

2. Department of Systemic Neurophysiology
Technische Universität Darmstadt
Schnittspahnstraße 3
64287 Darmstadt
Germany

3. Johann Wolfgang Goethe-Universität Frankfurt am Main
Faculty 15 Biological Sciences
Institute for Cell Biology and Neuroscience
Buchmann Institute for Molecular Life Sciences
Max-von-Laue-Str. 15
D-60438 Frankfurt am Main
Germany

4. Johann Wolfgang Goethe-Universität Frankfurt am Main
Institute for Forensic Medicine
Kennedy Allee 104
60596 Frankfurt am Main
Germany

* To whom all correspondence should be addressed

phone: +31 (0)43 38 82345
e-mail: sven.hildebrand@maastrichtuniversity.nl

phone: +49 6151 16-20950
e-mail: galuske@bio.tu-darmstadt.de

Keywords: human brain, hFRUIT, optical clearing, lipophilic tracer, DiI, PLP fixation

Supplementary Table 1. List of the different tissue sources and fixation types for every figure and video presented.

Figure	Tissue source/Animal	Fixation
1a,b; Supplementary figure 1	mouse	4% PFA
1c; 2; Supplementary figure 2+3	pig	PLP
3a; 5	body donor	formalin
3b; 4; Supplementary video 1+2	autopsy	PLP

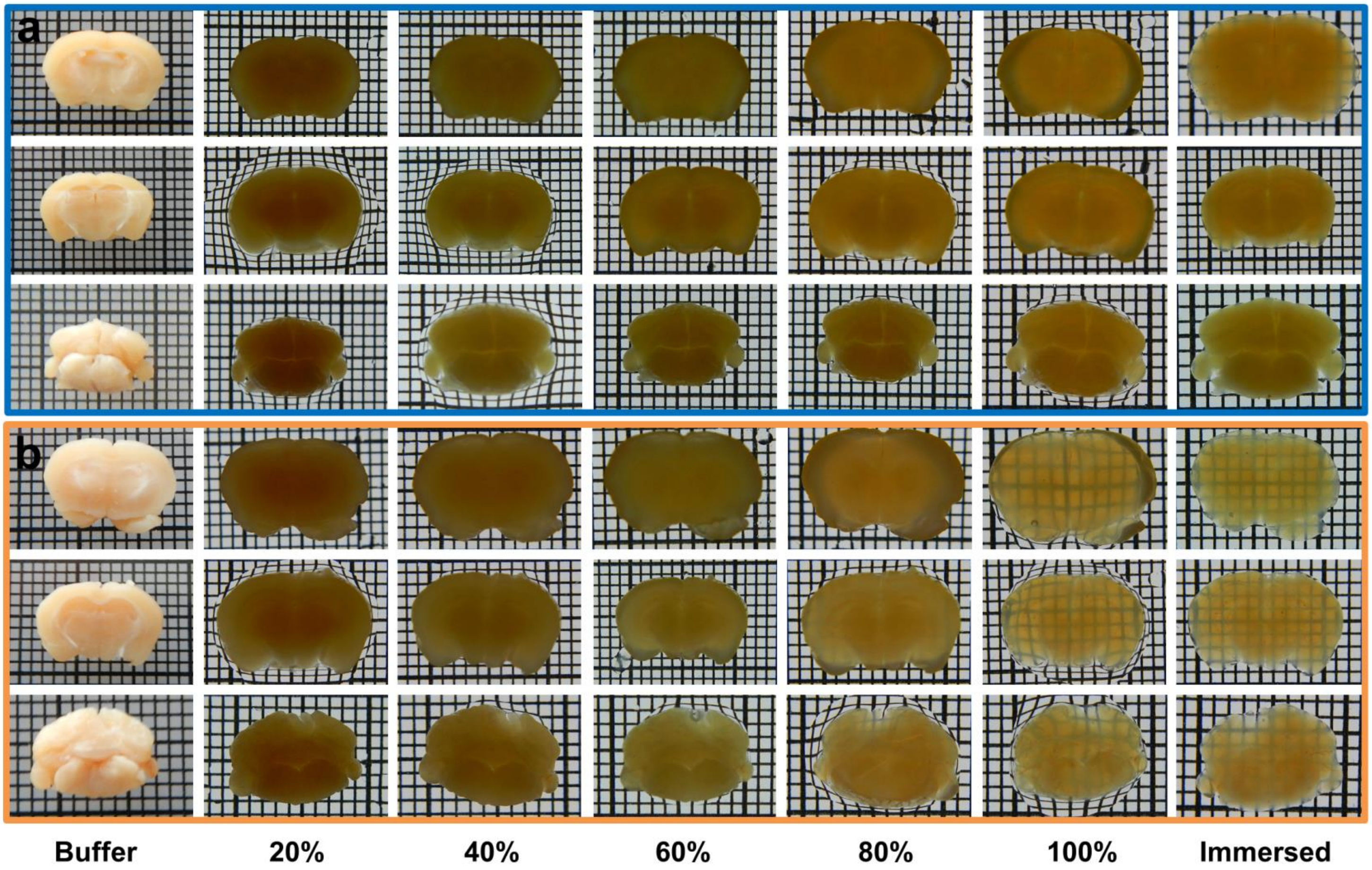

Supplementary Figure 1. (a) FRUIT clearing performance on 3 mm thick coronal slices of mouse brain and whole mouse cerebellum (bottom row) over ascending series of concentrations. (b) hFRUIT clearing performance on 3 mm thick coronal mouse brain sections and whole cerebellum (bottom row) for each concentration. Last column shows samples immersed in 1:1 mixture of silicon oil and mineral oil respectively. Grid size 1x1 mm respectively.

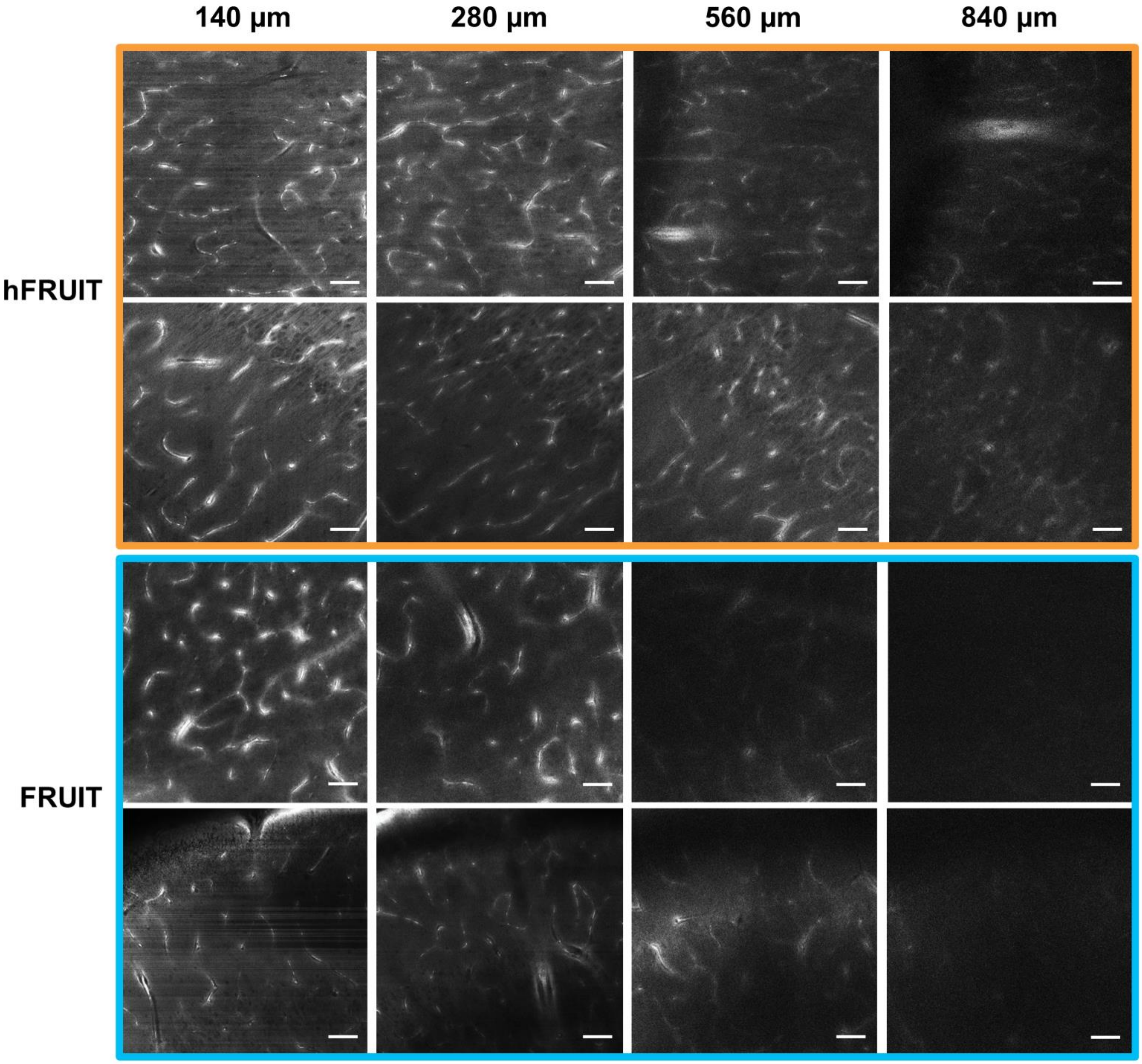

Supplementary Figure 2. Comparison of four representative porcine brain samples, two cleared with hFRUIT (orange) and two FRUIT (blue) respectively. Shown is a qualitative comparison of the autofluorescent blood vessel signal over increasing depths when excited with 488 nm, with each panel adjusted for contrast and brightness (not the signal drop-off over depth). Scale bars: 100 µm each.

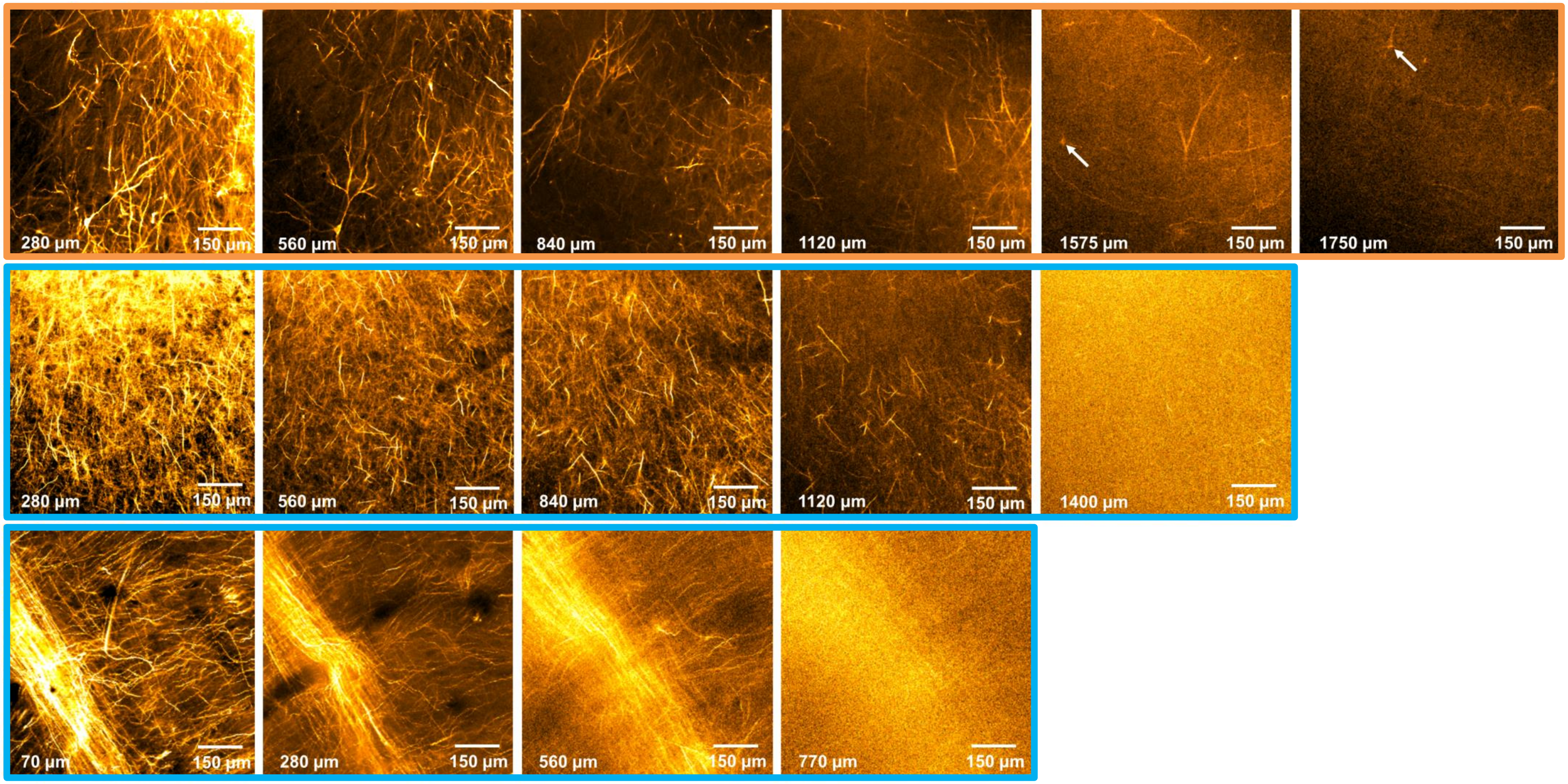

Supplementary Figure 3. Comparison of representative DiI-labelled porcine brain samples cleared with hFRUIT (orange box) or FRUIT (blue boxes) and imaged with CLSM. hFRUIT cleared specimen allowed for deeper imaging of structures in general, with cell bodies being visible up to at least 1500 µm.

Supplementary Video 1. Single sided x-scan of DiI-labelled human amygdala, cleared with hFRUIT and acquired with the diSPIM set-up. The injection site of DiI crystals is located in the upper left corner. Volume dimensions: 400x772x546 µm.

Supplementary Video 2. X-scan from the opposite direction of the same sample. The injection site of DiI crystals is located in the upper right corner. Volume dimensions: 400x772x546 µm.

Supplementary Video 3. 3D rendering of a deconvolved sub-volume of the data set shown in Supplementary Video 1 and 2.
